# Antioxidant effects of seaweeds and their active compounds on animal health and production – a review

**DOI:** 10.1080/01652176.2022.2061744

**Published:** 2022-04-08

**Authors:** Izabela Michalak, Ruchi Tiwari, Manish Dhawan, Mahmoud Alagawany, Mayada R. Farag, Khan Sharun, Talha Bin Emran, Kuldeep Dhama

**Affiliations:** a Faculty of Chemistry, Department of Advanced Material Technologies, Wrocław University of Science and Technology, Wrocław, Poland; b Department of Veterinary Microbiology and Immunology, College of Veterinary Sciences, Uttar Pradesh Pandit Deen Dayal Upadhyaya Pashu Chikitsa Vigyan Vishwavidyalaya Evam Go Anusandhan Sansthan (DUVASU), Mathura, India; c Department of Microbiology, Punjab Agricultural University, Ludhiana, India; d The Trafford Group of Colleges, Manchester, United Kingdom; e Poultry Department, Faculty of Agriculture, Zagazig University, Zagazig, Egypt; f Forensic Medicine and Toxicology Department, Veterinary Medicine Faculty, Zagazig University, Zagazig, Egypt; g Division of Surgery, ICAR-Indian Veterinary Research Institute, Bareilly, Uttar Pradesh, India; h Department of Pharmacy, BGC Trust University Bangladesh, Chittagong, Bangladesh; i Division of Pathology, ICAR-Indian Veterinary Research Institute, Bareilly, Uttar Pradesh, India

**Keywords:** Seaweeds, antioxidants, polysaccharides, animal production, health, diseases

## Abstract

Natural antioxidants applied as feed additives can improve not only animals’ health and overall performance but also increase their resistance to environmental stress such as heat stress, bad housing conditions, diseases, etc. Marine organisms, for example seaweeds – red, brown, and green macroalgae contain a plethora of biologically active substances, including phenolic compounds, polysaccharides, pigments, vitamins, micro- and macroelements, and proteins known for their antioxidant activity, which can help in the maintenance of appropriate redox status in animals and show pleiotropic effects for enhancing good health, and productivity. The dysregulated production of free radicals is a marked characteristic of several clinical conditions, and antioxidant machinery plays a pivotal role in scavenging the excessive free radicals, thereby preventing and treating infections in animals. Supplementation of seaweeds to animal diet can boost antioxidant activity, immunity, and the gut environment. Dietary supplementation of seaweeds can also enhance meat quality due to the deposition of marine-derived antioxidant components in muscles. The use of natural antioxidants in the meat industry is a practical approach to minimize or prevent lipid oxidation. However, overconsumption of seaweeds, especially brown macroalgae, should be avoided because of their high iodine content. An important point to consider when including seaweeds in animal feed is their variable composition which depends on the species, habitat, location, harvest time, growing conditions such as nutrient concentration in water, light intensity, temperature, etc. This review highlights the beneficial applications of seaweeds and their extracted compounds, which have antioxidant properties as feed additives and impact animal health and production.

## 1. Introduction

Seaweeds, also called macroalgae, are multicellular large-size marine organisms, which are classified into three main groups, according to their pigments – green (Chlorophyta), red (Rhodophyta), and brown seaweeds (Phaeophyceae) (Makkar et al. 2016; Corino et al. 2019; Alboofetileh et al. 2021). This biomass has been used for centuries in many coastal communities and played an important role as a component of food, natural fertilizer or animal feed (Mac Monagail et al. 2017). According to the latest Food and Agriculture Organization (FAO) report (2018), in 2015, the total world seaweed production was about 30.4 million tonnes – 29.4 million tonnes from aquaculture (seaweed cultivation) and 1.1 million tonnes harvested from the wild. Cultivation of seaweeds dominates in China, Indonesia, the Republic of Korea, and the Philippines, whereas the leading producers for wild species are Chile, China, and Norway. It is assumed that 221 species of seaweeds have a commercial value. Intensively cultivated are species of brown seaweeds – *Saccharina japonica*, *Sargassum fusiforme*, *Undaria pinnatifia*, red seaweeds – *Eucheuma* spp., *Gracilaria* spp., *Porphyra* spp., *Kappaphycus alvarezii* and green seaweeds such as *Caulerpa* spp., *Enteromorpha clathrata*, *Monostroma nitidum*. Among them, the most popular is *Saccharina japonica* (earlier classified as *Laminaria japonica*), which production constitutes 33%.

Seaweeds are known to be rich in both primary and secondary metabolites. The primary metabolites, which are directly involved in processes such as growth, development, reproduction to perform physiological functions, can be divided into several classes: carbohydrates (fucoidan, alginate, laminarin in brown seaweeds, agar, carrageenan in red seaweeds, and ulvan in green seaweeds), lipids (fatty acids: saturated, mono-, di-, and polyunsaturated, waxes, acylglycerols: mono-, di-, and triacylglycerols, phospholipids, glycoglycerolipids, fat-soluble vitamins, e.g., A and E, sterols, and carotenoids), and proteins (combination of different amino acids). In addition to the primary metabolites, seaweeds can accumulate minerals (microelements, macroelements, and trace elements) that are also essential to their life (Makkar et al. 2016; Salehi et al. 2019; Matos et al. 2021). Seaweeds can also synthesize a vast number of secondary metabolites, which largely determine their bioactive potential (Øverland et al. 2019; Salehi et al. 2019). In contrast to primary metabolites, secondary metabolites are not necessary for tissue growth. They are formed in the biomass as a response to exposure to biotic (e.g., fungi, bacteria, viruses, insects, etc.) and abiotic stress (e.g., UV radiation, drought, salinity, high temperature) (Metsämuuronen and Sirén 2019; Matos et al. 2021). An important group of seaweeds’ secondary metabolites are phenolics, which involve simple phenols like phenolic acids and polyphenols, including flavonoids and non-flavonoids – e.g., tannins. Carotenoids and sterols listed above as primary metabolites are often included in the secondary metabolite class (Salehi et al. 2019). Most of the listed compounds have antioxidant activity. Seaweed antioxidants play the function of “free radical scavengers” – they prevent or repair damages caused by oxidative stress and have a high potential for treating various diseases (Liu and Sun 2020). This unique composition of seaweeds results from their growth in an ever-changing environment. They are constantly exposed to stress conditions such as salinity, changes in temperature, nutrient enrichment, UV radiation exposure, presence of herbivores or pollutants. The resistance of seaweeds to biotic and abiotic stress is due to the production of physiologically active substances such as oligosaccharides, amino acids, vitamins, and phytohormones (Farvin and Jacobsen 2013; Manlusoc et al. 2019; Matos et al. 2021).

**Figure 1.**
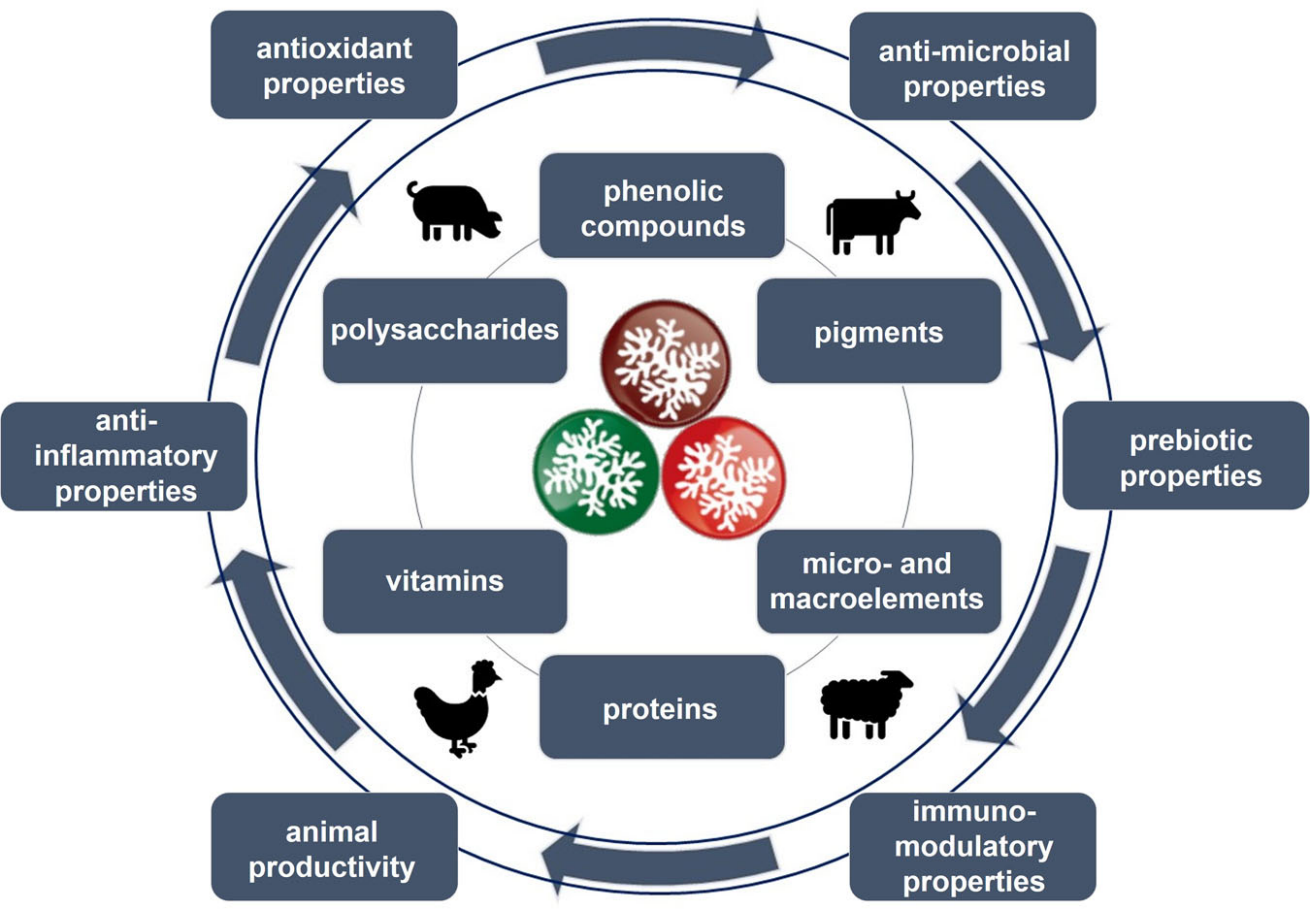
The general scheme of seaweed antioxidants and their effect on animal health and performance.

Seaweed-based antioxidants are discussed mainly in terms of cosmetic, pharmaceutical, and biomedical applications, food applications (stabilizer and preservative), and agriculture (biostimulants of plant growth) (Cotas et al. 2020), but less in terms of feed and animal health and production. Antioxidants derived from seaweeds can have many beneficial effects on animal health (de Quiros et al. 2010; Liu and Sun 2020), such as antioxidant, anti-inflammatory, antimicrobial, immunomodulatory, and prebiotic effects (Corino et al. 2019; Liu and Sun 2020). The supplementation of animal feed with prebiotics based on seaweed biomass has recently gained much attention due to their positive effect on gut health and immune system (Ruiz et al. 2018). The general scheme showing the seaweed antioxidants and their effect on animal health and performance is presented in Figure 1.

This review focuses on the application of seaweeds, seaweed extracts, and extracted compounds in animal feed. Special attention has been paid to antioxidants and their effects on animal health and production performance. Furthermore, the significance of seaweeds’ antioxidants, their different forms and use in animal nutrition, and their influence on the quality of animal-derived products have been discussed.

## 2. Seaweeds as a source of antioxidants

Seaweeds can serve as a source of antioxidants as they are produced by the biomass as a result of exposure to external environmental factors such as herbivory, salinity, nutrients availability, light, depth, seasonality as well as intrinsic factors such as age, length, and type of tissues (Farvin and Jacobsen 2013; Manlusoc et al. 2019). The main groups of antioxidants in seaweeds are phenolics, polysaccharides, and pigments.

Phenolic compounds found in seaweeds include (a) simple phenols such as phenolic acids (de Quiros et al. 2010): hydroxycinnamic acids – caffeic, *p*-coumaric, ferulic, sinapic acid and hydroxybenzoic acids – gallic, vanilic, 4-hydroxybenzoic, protocatechuic, syringic, gentisic acid (Farvin and Jacobsen 2013) and (b) polyphenols covering flavonoids and non-flavonoids. Flavonoids have several subgroups, which include flavones (e.g., rhoifolin), flavanol (e.g., catechins, epicatechin, gallocatechin, epigallocatechin), flavanones, flavonols (e.g., quercetin), anthocyanins (e.g., delphinidin, isopeonidin, malvidin), and isoflavones (e.g., sativanone, dalbergin) (de Quiros et al. 2010; Farvin and Jacobsen 2013; Cotas et al. 2020; Zhong et al. 2020). Non-flavonoids are divided into tannins (de Quiros et al. 2010; Cotas et al. 2020) such as phlorotannins (phloroglucinol and eckol) (Cotas et al. 2020), lignans (lignan derivatives: 2′-hydroxyenterolactone, arctigenin, dimethylmatairesinol, deoxyschisandrin), and stilbenes (Zhong et al. 2020). Phlorotannins are the major phenolic compounds in brown seaweeds, which are unique to this seaweed class (de Quiros et al. 2010; Holdt and Kraan 2011; Ford et al. 2020a). Other polyphenols which can be distinguished in seaweeds are phenolic terpenes (rosmanol, carnosol, carnosic acid [Zhong et al. 2020]) and terpenoids (chromene, chromanol, plastoquinone [Cotas et al. 2020]). Phenolic terpenoids were determined and characterized in red and brown seaweeds (Stengel et al. 2011). Seaweeds also contain bromophenols like 2,4-bromophenol, 2,6-bromophenol, 2,4,6 tribromophenol (de Quiros et al. 2010; Cotas et al. 2020). Besides antioxidant properties (prevention and/or reduction in oxidative damage), most phenolic compounds possess a wide variety of biological activities such as antibacterial, antiviral, anti-inflammatory, antidiabetic, antimethanogenic, etc. (Corino et al. 2019; Campbell et al. 2020; Cotas et al. 2020; Ford et al. 2020a). Phenolic compounds are considered an alternative to antimicrobials in feed, which can help reduce antibiotic resistance in livestock (Campbell et al. 2020; Ford et al. 2020b). It is suggested that the antibacterial activity of extract produced from brown seaweed (*Ascophyllum nodosum*) can be attributed to the presence of phlorotannins (Nagayama et al. 2002).

Furthermore, seaweeds are known to contain unique polysaccharides, which are specific to a given algal group. The content of carbohydrates in all seaweeds is recognized as high (Ford et al. 2020a; Matos et al. 2021). Øverland et al. (2019) presented the ranges of their content – brown seaweeds 380–610 g/kg of dry mass (d.m.); green seaweeds 150–650 g/kg d.m. and red seaweeds 360–660 g/kg d.m. Polysaccharides like fucoidan, alginate, laminarin are found in brown seaweeds, agar and carrageenan in red seaweeds, and ulvan in green seaweeds (Kraan 2012; Corino et al. 2019; Øverland et al. 2019; Liu and Sun 2020; Morais et al. 2020). Sulfated polysaccharides from marine algae such as fucoindan, carragenan, and ulvan possess antioxidative activity and can enhance the antioxidant status in animals (Salehi et al. 2019; Liu and Sun 2020). Seaweeds are rich not only in polysaccharides acting as antioxidants but also in the undigestible polysaccharides, thereby serving as a potential source of dietary fibers (O’Doherty et al. 2010). The dietary fibers are classified into two groups (1) insoluble such as cellulose, mannan, and xylan, and (2) water-soluble such as alginic acid, furonan, laminarin, agar, and porphyran (Kraan 2012). Polysaccharides applied as prebiotics in animal feed positively affect the cellulolytic and lactic acid bacteria present in the gastrointestinal tract (GIT) and inhibit the occurrence of pathogenic bacteria such as *Escherichia coli* and *Clostridium difficile*. Maintenance of the adequate gut microbiome and reduced risk of inflammation enhance gut morphology and nutrient absorption from the feed (Lynch et al. 2010; Kraan 2012). Polysaccharides, especially laminarin and fucoidan, have a positive effect on animal health – modulate the gut environment, stimulate the innate immune system, reduce the risk of diarrhea, enhance growth performance and promote productivity (also in the absence of in-feed antibiotics) (Lynch et al. 2010; Kraan 2012; Del Tuffo et al. 2019).

Natural pigments are another group of bioactive compounds with antioxidant properties (de Quiros et al. 2010; Holdt and Kraan 2011; Øverland et al. 2019; Matos et al. 2021). Seaweeds contain chlorophylls, mainly green seaweeds (de Quiros et al. 2010; Øverland et al. 2019; Morais et al. 2020), *β*-carotene, lutein, zeaxanthin, phycobiliproteins – red seaweeds (de Quiros et al. 2010; Øverland et al. 2019), carotenoids (α- and β-carotene, lutein, zeaxanthin, and fucoxanthin) – brown seaweeds (de Quiros et al. 2010; Farvin and Jacobsen 2013; Øverland et al. 2019; Morais et al. 2020), whereas fucoxanthin is typical to brown seaweeds (Cotas et al. 2020) and masks chlorophyll, β-carotene, violaxanthin and diatoxanthin (Corino et al. 2019).

Seaweeds also contain a wide array of vitamins, including vitamin E (α-tocopherol) (de Quiros et al. 2010; Farvin and Jacobsen 2013; Morais et al. 2020), vitamin C (ascorbic acid) (de Quiros et al. 2010; Morais et al. 2020; Matos et al. 2021; Nielsen et al. 2021), A, B_1_, B_2_, B_3_, B_5_, B_6_, and B_8_ (Corino et al. 2019; Morais et al. 2020). In addition, some carotenoids also act like vitamins, for example, β-carotene with the provitamin A activity (Holdt and Kraan 2011).

Micro- and macroelements (e.g., iodine, zinc, iron, manganese, selenium, sodium, calcium, potassium, and magnesium) are other seaweed constituents important for the proper development and health of animals. Seaweeds easily bioaccumulate minerals from seawater and contain 10–20 times more minerals than land plants (Makkar et al. 2016; Matos et al. 2021). Micro- and macroelements are present in a form, which is highly bioavailable to animals. Some microelements – e.g., selenium, zinc, and manganese demonstrate strong antioxidant properties (Corino et al. 2019; Øverland et al. 2019; Liu and Sun 2020; Morais et al. 2020). Feed for animals containing seaweeds are usually higher in ash than the feed/concentrates used in the control group (Rjiba et al. 2010). Seaweeds possess the ability to accumulate micro- and macroelements and toxic metal ions such as arsenic, cadmium, and lead, which can limit their use as a component of animal feed (Corino et al. 2019; Øverland et al. 2019). The same is the case of high iodine content (Øverland et al. 2019).

Seaweeds constitute a valuable source of proteins (Farvin and Jacobsen 2013; Makkar et al. 2016; Corino et al. 2019; Morais et al. 2020; Matos et al. 2021) with red seaweeds as the richest source of protein − 64–376 g/kg d.m. (crude protein), then green seaweeds 32–352 g/kg d.m. and finally brown seaweeds 24–168 g/kg d.m. (Øverland et al. 2019). Seaweeds can provide animals with all essential and many non-essential amino acids. Therefore, a detailed amino acid analysis of seaweeds should be performed for nutritional purposes (Øverland et al. 2019). Essential amino acids in *Sargassum latifolium* represent 42% of the total amino acids content (12 mg/g d.m.). Therefore, they are suggested as a complementary source of proteins for animal nutrition (Ramadan et al. 2020).

An important point to consider when including seaweeds in animal feed is their variable composition which depends on the species, habitat, location, time of harvest, growing conditions such as nutrient concentration in water, light intensity, temperature, etc. (Makkar et al. 2016; Del Tuffo et al. 2019). It is recommended to harvest seaweeds when active compounds are the most abundant to reduce the amount of algal biomass supplemented to the feed (Ford et al. 2020a). Therefore, the use of seaweeds in animal nutrition as a source of antioxidants should be preceded by a detailed analysis of the algae composition, depending on the species, location, harvest date, etc.

## 3. Significance of antioxidants to animals

Oxidative stress and increased production of reactive oxygen species (ROS) regulate several organs and tissues' metabolic activities under diverse veterinary conditions and play a significant role in the productive output of livestock. The oxidative damage caused by the increased generation of unnecessary ROS due to the activity of NADPH-oxidases results from the exposure to multiple stress factors, including biotic and abiotic factors (Kaur et al. 2014). In the pathophysiology of various infections and diseases, such as foot and mouth viral infection and interdigital dermatitis, the weakening of antioxidant defense has an essential role in the prognosis of the disease (Khoshvaghti et al. 2014; Hayat et al. 2020).

Enzymatic antioxidant machinery comprises catalase (CAT), glutathione peroxidase (GPX), and superoxide dismutase (SOD), and other enzymes (Kaur et al. 2016). At the same time, the non-enzymatic antioxidants include vitamins A, C, and E, thiol antioxidants (thioredoxin, glutathione (GSH) and lipoic acid), carotenoids, melatonin, natural flavonoids, etc. (Rahman 2007; Valko et al. 2007; Kurutas 2016; Changxing et al. 2018a, 2018b). Several biotic and abiotic stress factors (e.g., bacteria, environmental pollutants, radiation, chemicals, medications, food, diseases, and temperature) may influence the concentration of multiple antioxidant enzymes like CAT, GPX, SOD, and GSH, MDA (malondialdehyde – a product of lipid peroxidation in animal blood), as well as their activities. The reduction in the concentration of antioxidant enzymes and their activities increases free radical’s generation levels. The virulence effect of pathogenic microorganisms is heightened by the presence of elevated amounts of free radicals in macrophages, which are released in large numbers when bacterial infections arise. CAT, GPX, and GSH are essential components of the cell's mechanism of protection against oxidative stress, which help sustain the immune cells' functions against infectious diseases (Rahman 2007; Valko et al. 2007; Rahal et al. 2014).

Several studies in various clinical manifestations have explored the role of antioxidant enzymes and their functioning. A study was conducted to evaluate the relationship between antioxidant enzymes such as SOD, GPX, MDA, CAT, and foot and mouth disease (FMD) in cattle. Forty diseased cows affected with FMD virus were compared with ten healthy adult cattle as a control. SOD and GPX activities were significantly reduced by FMD virus infection as compared to the control group. MDA activity was significantly higher in infected animals compared with healthy cows. It has been reported that the antioxidant enzymatic machinery significantly reduced its functioning in viral infection in cattle, which postulates the possible roles of oxidative stress in clinical manifestation (Khoshvaghti et al. 2014). In another study, the correlation between acute bovine laminitis and antioxidant enzymatic activities of MDA, CAT, GSH, and SOD was measured in dairy heifers. A significant reduction in SOD concentration was recorded when compared to the control group, whereas MDA activities were significantly higher in treated heifers than in the healthy heifers. Non-significant differences were recorded in GSH and CAT activities between the control and experimental group. It has been postulated that inadequate levels of antioxidants (enzymatic and non-enzymatic) may be linked to oxidative stress in sick heifers. Antioxidant enzymes such as SOD, GSH, CAT, and MDA activity might play a crucial role in the pathogenesis of acute laminitis in dairy heifers (Hayat et al. 2020). Further, another study reported that the enhanced production of MDA and NO in the case of interdigital dermatitis infection. CAT levels were significantly lower in serum compared to the control group in several conditions, including interdigital dermatitis, footrot, and interdigital pouch infections. GPX and GSH concentrations in serum were also reported significantly lower in interdigital dermatitis infections. Further, it has been recorded that MDA and NO concentrations were significantly higher in sheep with foot disease whereas, CAT concentrations were significantly lower in serum. It has been postulated that there is a strong correlation between foot infectious diseases and antioxidant machinery in animals. Hence, oxidative stress and increased ROS levels lead to lipid peroxidation and protein and DNA damage, which enhance the severity of the infection (Yurdakul and Yildirim 2018).

Antioxidants in the body cells are present in small amounts, which positively limit the oxidation of important cellular components such as proteins, DNA or RNA. Additionally, natural or synthetic antioxidants may be used either endogenously or exogenously as a component in livestock diet or as a feed additive. Ascorbic acid, among other vitamins and nutrients, has been believed to be an antioxidant agent, which can reduce and neutralize reactive oxygen species. This vitamin, being a cofactor for several enzymes, is required for normal growth and development (Eryilmaz 2017; Nielsen et al. 2021). Seaweeds can serve as a source of vitamin C – the average content is 0.773 mg/g d.m. The highest mean value of this vitamin was observed in brown seaweeds − 0.815 mg/g, then green seaweeds − 0.781 mg/g, and finally in red seaweeds − 0.720 mg/g d.m. (Nielsen et al. 2021). Physiologically, strong antioxidants can be readily absorbed and effectively remove free radicals while also removing redox metals at appropriate amounts. The endogenous antioxidant serves a key role in helping the cells work more efficiently (Arain et al. 2018a, 2018b; Waheed Janabi et al. 2020; Naiel et al. 2021). Recently, it has been suggested that persistent oxidative stress may contribute to chronic inflammation, which in turn mediate a variety of chronic diseases in animals (Juárez-Portilla et al. 2019). For example, a link between tumor initiation and progression and oxidative stress has been demonstrated either by genetic mutations or DNA injury (Visconti and Grieco 2009). In comparison, the release of histamine or other inflammatory molecules and the increase in the inflammation-inducing white blood cells, like mast cells and neutrophils, resulting in a rise of the blood supply to the affected region, thereby enhancing the oxygen influx, which in turn leads to oxidative stress at the site of infection. Therefore, increased development and concentration of oxidative stress leads to the infection's severity in various clinical manifestations. Proinflammatory cytokines, as well as specific mediators of inflammatory cells, including the derivatives of fatty acids, cytokines, and chemokines, can also respond through growing the number of immune cells present at the injury or disease site and thereby augmenting the output of ROS (Coussens and Werb 2002; Hussain et al. 2003).

Besides, free radicals are frequently found in animals' neuropathological conditions. Hence, the defense against oxidative damage could serve as a means of preventing disease progression such as neurodegeneration in animals. Among other aspects, researchers found that ROS production is closely linked to inflammation, and marine algae compounds containing both antioxidant and anti-inflammatory properties may be excellent choices for treating hyperinflammatory responses in several animal diseases due to their complex and multifaceted properties (Barbalace et al. 2019). Barbalace et al. (2019), in their comprehensive literature survey, showed that seaweed chemical components such as antioxidants, fatty acids, polysaccharides, sterols, carotenoids, phycobilins, phycocolloids, etc. possess the potential in reducing proinflammatory cytokines such as NO, TNF-α, IL-6, and IL-1β and in downregulating the inflammatory enzymes such as iNOS and COX-2. Further, the antioxidant properties of several seaweed extracts have been reported to attenuate the signaling mechanisms contributing to inflammatory enzymes activation (Barbalace et al. 2019).

## 4. Forms of seaweeds’ antioxidants in animal nutrition

Seaweeds as a feed/feed additive can be used in the form of intact natural biomass (fresh or dried) or in the form of extract (Dierick et al. 2009; Michiels et al. 2012; Michalak and Mahrose 2020). The intact seaweeds are usually incorporated into feed pellets, whereas extracts into solutions (drinking water) (Abbott et al. 2020). The biomass after collection from the coastal zone is, in most cases, washed with tap water to remove sodium and impurities, dried, grounded, and finally used as a seaweed powder in animal diet (Abdoun et al. 2014; Ramadan et al. 2020). In this case, not only antioxidants but also other seaweed bioactive compounds are supplemented to animals. Campbell et al. (2020) proposed ensiling of seaweeds before application in livestock feeding, which is a suitable method of biomass preservation. Ensiling of brown seaweeds – *Saccharina latissimi* and *Fucus vesiculosus* with or without lactic acid bacteria inoculant (*Lactobacillus plantarum*) had a limited effect on the phenolic content. Fermentation for 90-days did not influence the chemical composition of *F. vesiculosus*, but for *S. latissimi* the content of crude protein, ash, and fiber was decreased.

To make seaweeds’ biomass a valuable source of antioxidants for animals, it should be subjected to the extraction process (Table 1). Before extraction, this biomass is usually pretreated (washing, drying, milling or grinding, pre-extraction) to obtain a higher yield of seaweed antioxidants (Cotas et al. 2020). The content of antioxidants in the produced extract depends on different extraction methods, as well as on the tested seaweed species (Farvin and Jacobsen 2013). Usually, seaweed extracts are a concentrate of many bioactive compounds. But appropriate extraction method can be selected aimed at extracting a given compound. For example, to extract polysaccharide – ulvan from green seaweed (*Ulva* sp.) used as a feed additive for laying hens, extraction with cellulase and later with hydrogen peroxide was applied (Li et al. 2018). However, the most appropriate method of seaweeds delivery to animals is not possible to predict at this moment. It will require animal feeding trials suitable for the animal production method used in a given country (Abbott et al. 2020).

**Table 1.** The examples of the effect of seaweeds antioxidants on animals.

Seaweed	Tested antioxidant/dose/animal	Effect on animals (as compared to the control group)	Reference
Polysaccharides			
*Laminaria digitata* (B)	extract containing laminarin − 0.5 g/kg feed and fucoidan − 0.42 g/kg feed, pigs	no effect on the plasma total antioxidant status, increase in antioxidant activity in animals – lowering levels of lipid oxidation in porcine muscle	Moroney et al. 2012
*Laminaria digitata* (B), Bioatlantis Ltd (Tralee, Ireland)	extract containing laminarin (112 g/kg) and fucoidan (89 g/kg), dose 2.8 g/kg, newly weaned pigs	improved performance, higher average daily gain, gain to feed ratio, increase in the coefficient of total tract apparent digestibility of N, gross energy, reduction in the counts of *E. coli* in faeces	O’Doherty et al. 2010
*Laminaria hyperborea* (B) *Laminaria digitata* (B)	extract containing laminarin and fucoidan, 1.5 g/kg, weaned pigs	*L. hyperborea* extract – lower population of *Bifidobacteria* in the colon and *Lactobacilli* in the caecum and colon, increase in total monocyte number, *L. digitata* extract – lower population of *Enterobacteria* in the caecum and colon, *Bifidobacteria* in the caecum, *Lactobacilli* in the caecum and colon, for both extracts – reduced ammonia concentration in the colon	Reilly et al. 2008
*Laminaria* spp. (B), Bioatlantis Ltd (Tralee, Ireland)	extract containing laminarin (112 g/kg) and fucoidan (89 g/kg), dose 1, 2, 4 g/kg, weanling piglets	at low (60 g/kg) and medium (150 g/kg) levels of lactose in diet – increase in average daily gain with the increase in extract to 2 g/kg, at low level of lactose – significant improvement in food conversion ratio as the levels of seaweed extract increased to 4 g/kg, for medium level – improvement till 2 g/kg of extract	Gahan et al. 2009
*Laminaria* spp. (B)	extract containing laminarin and fucoidan, 300 mg/kg of laminarin, 240 mg/kg of fucoidan, mixture of 300 mg/kg of laminarin and 240 mg/kg of fucoidan, weanling piglet	laminarin – increase in daily gain and gain-to-feed ratio, increase in faecal dry matter, reduced diarrhea, reduced faecal *E. coli*, fucoidan – increase in *Lactobacilli*, no effect of fucoidan on faecal *Lactobacilli* when laminarin was added, a combination of laminarin and fucoidan reduced post-weaning diarrhea	McDonnell et al. 2010
*Laminaria hyperborea* (B)	extract containing laminarin and fucoidan, 0.7, 1.4, 2.8, 5.6 g/kg, pigs	increase in urine output, water intake with an increase in extract inclusion, effect on colonic *Bifidobacterium* spp. (higher than in control, decrease with a rise in extract dose) and on caecal *Enterobacterium* spp. (higher for 0.7 g/kg than in the control), decrease in caecal *Bifidobacterium* spp. and colonic *Lactobacilli* spp. as the level of seaweed extract increased	Lynch et al. 2010
*Laminaria hyperborea* (B)	extract containing laminarin and fucoidan, 300 mg/kg of laminarin, 240 mg/kg of fucoidan, mixture of 300 mg/kg of laminarin and 240 mg/kg of fucoidan, pigs	laminarin – reduced *Enterobacterium* spp. compared to the control diet, the combination of laminarin and fucoidan increased *Enterobacterium* spp. compared with alone polysaccharides, fucoidan – increased *Lactobacilli* spp. in the proximal colon and distal colon as compared with non-fucoidan diet	Lynch et al. 2010
*Laminaria* spp. (B), Bioatlantis Ltd (Tralee, Ireland)	seaweed extract containing laminarin (1.0 g), fucoidan (0.8 g), and ash (8.2 g), sows and post-weaned pigs, 2.8 g/kg diet	higher average daily gain (0–21 post-weaning day) in pigs weaned from extract-supplemented sows, increase in average daily gain during the grower–finisher phase, decreased colonic *E. coli* population in pigs feed with post-weaning diets with extract	Leonard et al. 2011a
*Laminaria* spp. (B), Bioatlantis Ltd (Tralee, Ireland)	seaweed extract containing laminarin (1.0 g), fucoidan (0.8 g), and ash (8.2 g), 10 g/d, suckling piglet	increase in immunoglobulin G (IgG) and immunoglobulin A (IgA) in sow colostrum, an increase in piglet serum IgG, reduction in fecal *Enterobacteriaceae* numbers in sows at parturition, decrease in colonic *E. coli* population at weaning in piglets suckling extract-supplemented sows	Leonard et al. 2012
*Ascophyllum nodosum* (B), Tasco-14 (Acadian, Nova Scotia, Canada)	2%, steers and heifers	antimicrobial properties, reduction in the prevalence of enterohemorrhagic *E. coli* on hide swabs and in fecal samples, possible inhibition of the growth of *Salmonella* spp.	Braden et al. 2004
*Ascophyllum nodosum* (B), Tasco (Acadian, Nova Scotia, Canada)	extract, 1.7 and 3.4 kg/ha of fescue, grazing lambs	Increase in antioxidant activity in grazing ruminant, for summer lambs grazing period – increase in lamb gain, serum vit. A and Se concentration with increase in Tasco dose, no effect on vit. E	Fike et al. 2001
*Ascophyllum nodosum* (B), Tasco-Forage (Acadian, Nova Scotia, Canada)	tall fescue pastures infected with endophyte (*Neotyphodium coenophialum*) treated with Tasco-Forage (3.4 kg/ha), weaned beef steers	increase in rectal temperatures due to endophyte infection, decrease in temperature of steers grazing infected fescue treated with Tasco, alleviation of rough hair coats and loss of hair color, mitigation of the adverse effects of endophytes on immune function	Saker et al. 2001
*Ascophyllum nodosum* (B), Tasco^TM^ (Acadian Sealants Ltd, Nova Scotia, Canada)	wether lambs, heat stress	supplementation of Tasco to post-harvest fescue hay – enhancement of immune function (increased phagocytic activity, increase in red and white blood cell glutathione peroxidase), protection against prolonged heat-induced oxidative stress, enhancement of monocyte oxidative burst through short and long duration heat stress	Saker et al. 2004
*Ascophyllum nodosum* (B)	intact dried biomass – source of dietary fiber, 2.5, 5, 10 g/kg, weaned piglets	no significant effect on daily weight gain, feed conversion ratio, performances of piglets, gut health parameters and plasma oxidative status (measurements of TBARS, FRAP, GSH-Px), increase in plasma α-tocopherol in all experimental groups, no changes in the microbial ecology in the foregut and in the caecum	Michiels et al. 2012
*Ulva armoricana* (G)	algal extract containing sulfated polysaccharide, 2, 8 and 16 g/day (two periods: before the last atrophic rhinitis vaccine booster and a week before farrowing), pig, sows – the end of gestation	16 g/day – an increase in specific IgG in sow’s blood and colostrum, 8 g/day – an increase in the level of total IgA in milk as compared to the control group	Bussy et al. 2019
brown seaweeds (Dalian Institute of Chemical Physics, Chinese Academy of Sciences, Dalian, China)	alginic acid oligosaccharide, 100 mg/kg, weaned pigs	enhancement of the average daily body weight gain, increase in the concentrations of IL-10, IgG, and IgA, increase in SOD, CAT activity, and total antioxidant capacity in the serum, decrease in serum MDA, increase in the population of *Bifidobacterium* and *Lactobacillus*, decrease in the populations of *E. coli* in the intestine	Wan et al. 2016
brown seaweeds (Dalian Institute of Chemical Physics, Chinese Academy of Sciences, Dalian, China)	alginate oligosaccharide, 50, 100 and 200 mg/kg, weaned pigs	100 and 200 mg/kg – significant increase in the average daily body weight gain, 100 mg/kg – promotion of antioxidant defense properties (enhanced serum CAT activity and GSH content), improvement of serum hormone levels (insulin and insulin-like growth factor-1), increase in the nutrient digestibility (crude ash, protein, and fat), maltase and sucrase activities in the duodenal and jejunal mucosa	Wan et al. 2017
brown seaweeds (Dalian Institute of Chemical Physics, Chinese Academy of Sciences, Dalian, China)	alginate oligosaccharide, 100 mg/kg, weaned pigs	enhanced the intestinal integrity, increase in intestinal occludin protein abundance, increase in the jejunal and ileal catalase activity, decrease in the duodenal and jejunal tumor necrosis factor-α concentration, inhibition in the pro-inflammatory cytokines production	Wan et al. 2018
*Sargassum latifolium* (B)	seaweed powder containing mainly carbohydrates (41.4%), ash (26.2%), amino acids, phenolic compounds (carotenes, flavonoids, kaempherol, alkaloids), 2 and 4%, bacterial endotoxin (bacterial lipopolysaccharides)-challenged sheep	increase in the thermo-respiratory response (skin and rectal temperatures, respiration rate), the obtained systemic inflammation (blood leukocytosis, the increase in the erythrocyte sedimentation rate, in serum concentrations of proinflammatory cytokines, heat shock protein-70), improvement of the total antioxidant capacity of the blood (increase in the CAT and SOD activity), decrease in the blood markers of tissue damage (MDA concentration and the activity of alanine aminotransferase and lactate dehydrogenase)	Ramadan et al. 2020
*Sargassum latifolium* (B)	seaweed powder containing mainly carbohydrates (41.4%), ash (26.2%), amino acids, phenolic compounds (carotenes, flavonoids, kaempherol, alkaloids), 2 and 4%, environmental heat stress-induced toxicity, sheep	increase in the thermo-respiratory responses (skin and rectal temperatures, respiration rate) and the resulted dyslipidemia, anemia, systemic inflammation (blood leukocytosis, the increase in the erythrocyte sedimentation rate, in serum concentration of proinflammatory cytokines, heat shock protein-70), significant improvement of the body-weight gain, kidney functions (especially for 4%), blood antioxidant defense system (total antioxidant capacity, the activity of CAT and SOD), protection of animals from oxidative tissue damage and the risk of atherosclerosis	Ellamie et al. 2020
OceanFeed Swine® (mixture of green, brown and red seaweeds)(Milltown, Ireland)	carbohydrates (43%), also proteins (8%), vitamins and minerals, 5 g/kg, nursery and fattening pigs	improvement of average daily weight gain and feed efficiency, increase in the slaughter weight, a reduction of *E. coli* CFU, increase in *Lactobacillus* sp. in faeces samples	Ruiz et al. 2018
OceanFeed Swine® (mixture of green, brown and red seaweeds)(Milltown, Ireland)	carbohydrates (43%), proteins (8%), vitamins and minerals, 0.5% in gestation and 0.66% in lactation, 0.75% in nursery diet, sows and their offspring	no statistically significant impact on sow body weight during gestation and lactation, no differences in colostrum yield, composition of colostrum and milk, no effect on growth performance during nursery period, lower number of pathogenic bacteria (*Fusobacteriaceae*) for pigs from sows and higher of beneficial bacteria (*Ruminoccocaceae* and *Lachnospiraceae*)	Del Tuffo et al. 2019
Phenolic compounds			
*Ascophyllum nodosum* (B), *Fucus serratus* (B)	phlorotannin extract (0.1, 0.781, 1.56, 3.125, 20, 50 mg/mL) and whole seaweeds (1, 3, 5, 10, 20%), *in vitro* (pig digestibility model)	phlorotannin extract – significant decrease in the digestibility of feed for both seaweeds, smaller decrease for whole seaweeds (no significant difference was observed for inclusion rates till 5%), difference in digestibility for the same species and inclusion rate, but collected from different seasons (effect of seasonality on chemical composition)	Ford et al. 2020a
Vitamins			
*Ascophyllum nodosum* (B) (Kerry Enhancer, Kerry Algae, Curraheen, Tralee Co. Kerry, Ireland)	extract, vitamins E, K, A, B_1_, B_2_, B_12_, niacin and elements: Cu, Fe, Mn, I, Zn, Se, Ca, Mg, Na, K (3, 6 and 9 g/kg), grower–finisher pigs	increase in the dose of extract resulted in reduced daily gain, carcass weight, and kill-out yield, increase in adherent *Lactobacilli* in the colon but decrease in caecal *Bifidobacteria*, decrease in ileal coliform counts, no effect of treatment on feed intake, feed conversion ratio or carcass characteristics, intestinal pH	Gardiner et al. 2008
Microelements			
*Ulva rigida* (G), *Sargasum muticum* (B), *Saccorhiza polyschides* (B)	I in the biomass, 100 g/animal per day, Holstein Friesian lactating cows	improvement of the mineral status of dairy cattle, especially I and Se, increase in the concentration of Co, Cr, Fe, I, Se, and Zn in milk	Rey-Crespo et al. 2014
*Chaetomorpha linum* (G)	Zn, Mn, Fe, Ca and Na in the biomass, 20%, lambs fattening during drought periods	feed conversion ratio was higher than for the control group, no effect on organic matter digestibility, faecal N, urinary N, and N retention, lower final body weight	Rjiba et al. 2010
*Ulva* spp. (G)	seaweed meal (ash 375 g/kg d.m., crude protein 176 g/kg d.m., crude fat 339 g/kg d.m., neutral detergent fibre 322 g/kg d.m., 171 g/kg d.m. acid detergent fibre), 20, 25, 30 and 35 g/kg, hens	increase in feed intake, overall body weight gain, no effect on feed conversion eff ciency, lack of improvement of apparent nutrient digestibility, no dietary effects on serum biochemical indices (glucose, urea, phosphorus, calcium, total protein, albumin, bilirubin, amylase, creatinine, globulin)	Nhlane et al. 2020
*Ascophyllum nodosum* (B)	iodine-rich intact seaweed, 10 and 20 g/kg, weaned piglets	beneficial effect on microbial population – reduction in *E. coli* load in the stomach and small intestine, enhancement of *Lactobacilli*/*E. coli* ratio in the small intestine, increase in iodine content in several tissues in piglets	Dierick et al. 2009
*Ascophyllum nodosum* (B) kelp meal (Thorvin Inc., New Castle, VA)	seaweed meal iodine rich (820 mg/kg d.m.), 57, 113, 170 g/d, early lactation dairy cows	no effect of seaweed on milk yield, concentration of milk components (fat, protein, lactose, milk urea N), serum concentrations of thyroxine and triiodothyronine, linear increase in concentration of milk I with increased dose of seaweed, reduction in the plasma concentration of non-esterified fatty acids	Antaya et al. 2015

Where: B – brown, G – green seaweed

There are several methods used for the extraction of antioxidants such as Soxhlet, maceration, percolation, microwave-, ultrasounds-, enzyme-assisted extraction, subcritical, and supercritical fluid extraction, accelerated extraction, etc. (Cotas et al. 2020; Liu and Sun 2020; Monteiro et al. 2020; Matos et al. 2021). The simplest extraction methods can be applied for the isolation of antioxidants, such as maceration or shaking of seaweeds. Considering the potential use of algal extracts in animal nutrition, water as a solvent may be recommended. In the literature, for the extraction of phenolic compounds from seaweeds, usually, polar solvents are used, mainly aqueous mixtures of ethanol, methanol, acetone, or ethyl acetate (Monteiro et al. 2020). For example, Hwang and Thi (2014) compared the extraction method and antioxidant compound content in extracts obtained from edible red seaweed – *Porphyra tenera*. The extraction yield for water as a solvent at 37 °C was 25.5%, and 70% ethanol at 37 °C − 17.9%. The total phenolic content from water extraction (28.7 ± 0.5 mg GAE/g extract) was comparable to the ethanolic extract (30.2 ± 0.4 mg GAE/g extract). Phlorotannins from brown seaweeds can be extracted with a mixture of acetone and water (Ford et al. 2020a). As the main component in seaweeds, polysaccharides can be readily extracted with water or acetone (Liu and Sun 2020). Alginate oligosaccharides, which have great potential as a novel feed supplement, are produced via depolymerization of alginic acid polysaccharides from brown seaweeds using alginate lyases (Wan et al. 2016; Wan et al. 2017; Wan et al. 2018).

One seaweed preparation with antioxidant properties is Tasco®, produced by Acadian AgriTech™ (Dartmouth, Nova Scotia, Canada). This product is available in two forms: Tasco-Forage and Tasco-EX. The first one is used as an extract applied to foliage, which is grazed by livestock, and the second is used for direct feeding. Both forms are responsible for antioxidant responses measured in livestock (Allen et al. 2001; Saker et al. 2001; Saker et al. 2004).

The key issue in the use of seaweeds as animal feed/feed additive is their composition (especially the content of toxic elements that easily accumulate in the biomass, but also bacterial load, pathogens, mycotoxins, pesticides, dioxins, polychlorinated biphenyls) and palatability for the animals (seaweed supplement is usually given to animals in order to verify the feed acceptance) (Dierick et al. 2009; Michiels et al. 2012; Rey-Crespo et al. 2014). In addition, pelleting is recommended to avoid the rejection of seaweeds added to the feed by animals (Rjiba et al. 2010).

## 5. The effect of seaweed antioxidants on animal health and production

Table 1 presents examples of the effect of seaweed antioxidants on animal health and productivity. The antioxidant effect of seaweeds can be evaluated by measuring the total antioxidant capacity, the activity of primary antioxidant enzymes (superoxide dismutase, glutathione, and catalase), and the level of malondialdehyde (Wan et al. 2016; Corino et al. 2019; Liu and Sun 2020; Ramadan et al. 2020). As shown in Table 1, the inclusion of seaweeds in the animal diet can help maintain the appropriate redox status in animals, good health, and productivity. In addition, supplementation of seaweeds can boost antioxidant activity, immunity, and the gut environment. However, this effect has not been observed in all animal studies performed (it depends on the seaweed species, their doses in the diet, animal species, etc.).

Seaweeds and their active compounds can enhance growth performance (O’Doherty et al. 2010; Leonard et al. 2011a, 2011b) and increase the average daily body weight gain of animals (Wan et al. 2016; Wan et al. 2017; Ruiz et al. 2018; Ellamie et al. 2020; Nhlane et al. 2020). Brown seaweeds’ polysaccharides like alginate, laminarin, fucoidan, being soluble dietary fiber, increase feed intake (Allen et al. 2001). This effect was not confirmed in green seaweeds (*Ulva lactuca*) supplemented to the diet of lambs reared under heat stress conditions. This inclusion did not show any beneficial effect on body weight gain or feed conversion efficiency (Abdoun et al. 2014). The species of algae thus plays a key role in its impact on animal health and performance.

There are several seaweed-based preparations on the market like OceanFeed® (Milltown, Ireland) designed for nutrition of swine, equine, and bovine. For example, OceanFeed® Swine brings many benefits for gestating and lactating sows, post-wean pigs, and growing-finishing pigs – improved feed intake, feed conversion ratio, digestive function, nutrient use, enhanced performance, weight gain, gut health, and reduce diarrhea in pigs (Ruiz et al. 2018; Del Tuffo et al. 2019).

Finally, seaweed feed additives can biofortify animal products with algal active compounds (Dierick et al. 2009; Moroney et al. 2012; Rey-Crespo et al. 2014; Moroney et al. 2015), improve carcass quality, and prolong the retail shelf life (Braden et al. 2007; Moroney et al. 2015). Dietary supplementation of laminarin and fucoidan extracted from *Laminaria digitata* resulted in enhanced pork meat due to deposition of marine-derived bioactive antioxidant components in *longissimus thoracis et lumborum* muscle (Moroney et al. 2015). Moroney et al. (2012) found that the use of seaweed extracts containing fucoidan and laminarin reduced lipid oxidation to the minimum level in liver tissue homogenates. Thus, the use of natural antioxidants in the meat industry is a practical approach to minimize or prevent lipid oxidation (Moroney et al. 2015).

### 5.1. The main seaweed antioxidants examined in animal feeding

As shown in Table 1, mainly brown seaweeds were used in animal feeding as a source of antioxidants. Their main representatives were *Ascophyllum nodosum*, *Sargassum* sp. and *Laminaria* sp. The antioxidant function is played dominantly by polysaccharides – laminarin, fucoidan, and alginate. It is hypothesized that these polysaccharides positively influence the gut environment, immunoglobulins output, and the growth performance of animals (Del Tuffo et al. 2019).

The beneficial effect of *Ascophyllum nodosum* extract on animals is attributed to its chemical composition. Dierick et al. (2009) showed that the dried intact *A. nodosum* contains 503 g/kg of the total dietary fiber, whose main components are galactose 6.9 g/kg, xylose 16.8 g/kg, mannose 38.4 g/kg, glucose 44.8 g/kg, fucose 66.0 g/kg, uronic acids 144.4 g/kg and klason lignin 185.8 g/kg. These compounds have an additional prebiotic effect. *Ascophyllum nodosum* biomass also contains crude ash 211 g/kg, crude protein 49.0 g/kg, crude fat 50.0 g/kg, and iodine at the 400 mg/kg level. *Ascophyllum nodosum* extract is also a source of minerals and vitamins in animal diet – micro- and macroelements (Se 2–3 mg/kg, Mn 15–30 mg/kg, Cu 20–45 mg/kg, Zn 50–170 mg/kg, I 250–500 mg/kg, Fe 250–1000 mg/kg, Mg 2–5 g/kg, Ca 15–20 g/kg, Na 20–40 g/kg, K 110–130 g/kg), as well as vitamins (B_12_ < 1 mg/kg, B_1_ 0.1–0.3 mg/kg, B_2_ 4–8 mg/kg, K 4–9 mg/kg, niacin 8–25 mg/kg, A 20–40 mg/kg, E 100–250 mg/kg) (Gardiner et al. 2008). Many microelements derived from *Ascophyllum nodosum* biomass like Cu, Fe, and Zn are known to participate in animal antioxidant responses (Allen et al. 2001).

Another representative of brown seaweeds is *Sargassum latifolium* having a significant amount of carbohydrates (41.4%), ash (26.2%), and essential amino acids, which represent 42% of the total amino acids content (12 mg/g d.m.). Therefore, this alga is considered as a complementary source of protein in animal feed. Flavonoids, kaempherol, and carotenes are the main phenolic compounds in this biomass (Ramadan et al. 2020).

The popularity of Tasco**®** application (Acadian AgriTech™, Dartmouth, Nova Scotia Canada) in animal feeding results from its rich composition, which involves polysaccharide and oligosaccharide alginate 52% (alginic acid 25%, mannitol 5%, laminarin 3%, fucose containing sulfated polysaccharide 15%, other carbohydrates 4%), other proximate constituents like crude fat min 2%, crude protein 6%, crude fibre 6%, minerals – macro (P 0.1–0.2%, Mg 0.5–1%, Ca 1–3%, S 2–2.3%, K 2–3%, Na 2.4–4%) and microelements (Se < 1 mg/kg, Cu 4–15 mg/kg, Mn 10–50 mg/kg, Zn 35–100 mg/kg, I < 1000 mg/kg). It can contain toxic metals like As but below 3 mg/kg. Ash content in this preparation is about 22%. Tasco^®^ also provides animals with amino acids (in g/100 g of protein): cystine trace amounts, methionine 0.7, tyrosine 0.9, histidine 1.3, phenylalanine 2.3, proline 2.6, isoleucine 2.8, threonine 2.8, serine 3, valine 3.7, leucine 4.6, lysine 4.9, glycine 5, alanine 5.3, aspartic acid 6.9, arginine 8, glutamic acid 10 (Allen et al. 2001; Fike et al. 2001). This product is known to enhance immune function and improve carcass characteristics (Allen et al. 2001).

Popular products based on seaweed biomass are OceanFeed® Swine, OceanFeed® Equine, OceanFeed® Bovine, produced by Ocean Harvest Technology Company Ltd. (Tuam, Co Galway, Milltown, Ireland). These products contain multiple green, brown and red seaweeds species and constitute a 100% natural macroalgal blend. The proximate composition of OceanFeed^TM^ products (14.5% moisture) includes carbohydrates 43.2%, ash 30.4%, protein 8.0%, fibre 4.88% and fat 0.35%. The main vitamins are vit. C 80 mg/kg, niacin 6.3 mg/kg, vit. E 4 mg/kg, A 0.86 mg/kg, K_3_ 0.3 mg/kg, thiamine 0.1 mg/kg, as well as folic acid 165 μg/kg, biotin 21.8 μg/kg and cyanocobalamin 1.4 μg/kg. This preparation contains also a wide range of minerals such as S 5.0%, Cl 4.0%, Ca 2.8%, K 2.7%, Na 2.3%, P 1.22 g/kg, Fe 1440 mg/kg, Mn 715 mg/kg, I 64 mg/kg, Zn 15.2 mg/kg, Cu 5.1 mg/kg, Mg 2.15 mg/kg and Se 0.13 mg/kg.

### 5.2. Effect of seaweed antioxidants on animal health and infectious diseases

Seaweeds and their active molecules with antioxidant and immunomodulatory activities can reduce oxidative damage, which might play a crucial role in the prognosis and amelioration of several infectious diseases, thereby safeguarding animal and human health (Shi et al. 2017; Juárez-Portilla et al. 2019). In addition, the biologically active compounds function as modulators of various cellular signaling mechanisms implicated in a plethora of infectious diseases (Juárez-Portilla et al. 2019). Furthermore, distinct extracts from different seaweeds can promote growth efficiency and gastrointestinal tract health by changing its anatomy and thus enhancing the intestinal absorption and utilization of nutrients and by modifying intestinal flora and attenuating the immune system and hence strengthening the gut structural integrity (Reilly et al. 2008; McDonnell et al. 2010; Sweeney et al. 2012).

Seaweeds have been found as a beneficial diet supplement as they enhanced pig growth and improved the digestibility of animal feed due to the content of bioactive phytochemicals (Corona et al. 2017). Further, the efficacy of the seaweeds diet was linked with its antioxidant compounds as seaweed can enhance the immune system functioning by reducing oxidative damage. The highest content of phenols and phlorotannins (up to 12–14%) is observed in brown seaweeds – *Ascophyllum nodosum*, *Sargassum* spp., and *Fucus* spp. In green and red seaweeds, this content is below 1% (Holdt and Kraan 2011).

Based on the data collected in Table 1, it can be concluded that seaweeds with antioxidant compounds used in feed improved mainly gut health and enhanced the function of the immune system. Seaweeds’ polysaccharides can improve the composition of animal’s gastro-intestinal microbiota without disrupting their performance (Reilly et al. 2008). Laminarin and fucoidan extracted from *Laminaria* sp. may provide a dietary means to improve gut health as these polysaccharides with antioxidant and antimicrobial properties can reduce the intestinal population of *Enterobacterium* spp. and increase the population of *Lactobacilli* spp. (Lynch et al. 2010; McDonnell et al. 2010; O’Doherty et al. 2010). Gahan et al. (2009) suggested that the extract obtained from *Laminaria* spp., rich in laminarin and fucoidan, can be used as a substitute for lactose in piglet diets, being the substrate for microbial fermentation in the large intestine. This approach could reduce the level of lactose in the diet and maintain post-weaned piglet performance fed with the diet free from growth promoters. In addition, extracts from *Laminaria hyperborea* and *L. digitata*, containing laminarin and fucoidan reduced *Enterobacteria*, *Bifidobacteria*, and *Lactobacilli* in the caecum and colon of the weaned pigs (Reilly et al. 2008).

Recently, brown seaweed-derived laminarin has been shown to possess a considerable level of antioxidant activity (Rajauria et al. 2021), which directly affected gut mucous membranes or gut-associated lymph nodes and lymphoid tissues, improving the integrity of the gut and stimulating the immune function of the digestive tract. Further, it has been reported that dietary laminarin enhanced the expression of several genes encoding mucin production while feeding, specifically MUC2, an important mucin-producing gene in the intestine, resulting in increased mucin production in the digestive tract (Ryan et al. 2010). Laminarin is processed by the GIT’s cells and then transferred to certain immunologically important cells such as dendritic cells. Thus, it works on cytokine output to influence the digestive system employing an immunomodulatory effect. In other studies, it has been reported that the diet augmentation with laminarin and other extracts resulted in the downregulation of developing a network of pro-inflammatory chemokines and cytokines in intestinal and hepatic cells (Sweeney et al. 2012; Walsh et al. 2013). The ability to lower the levels of pro-inflammatory cytokines can enable the animal to receive more nutrients and thus develop at a greater rate by distributed nutrients apart from strengthening the immune system (Walsh et al. 2013). Because of these immunomodulatory impacts, one might predict that they may regulate hyperinflammatory responses in various autoimmune disorders and viral infections.

The second crucial polysaccharide of brown seaweeds – fucoidan, is a critical immunomodulatory compound with many biological functions that have not yet been completely understood. However, it was discovered that the diet supplemented with a consortium of two different macroalgae-derived compounds, including fucoidan, laminarin, and their mixture to pregnant sows, enhanced the antibodies production rate in the breast milk, which, in turn, increased the IgG concentrations in the new-born piglets' serum. The fecal enterobacterial levels in sows shortly before parturition were significantly lower, and an additional observation confirmed the reduced number of coliform bacteria in the suckling pigs, which was correlated with piglets dramatically increasing the length of villi compared to the number of crypts in the upper small intestine, as well as increased growth rate. It is possible that reduced numbers of *Escherichia coli* and greater pathogen effects in the intestinal tracts of weaned piglets can be driven by the accumulation of mammary laminarin of low-molecular-weight in piglets' gastrointestinal tract. Additionally, algal extract intake induces enhanced TNF and TFF (trefoil factor) production in the gastrointestinal tract, which indicates their beneficial effects in newborn pigs (Leonard et al. 2011b). Moreover, laminarin supplementation to the diet of pregnant sows following by infection of *Salmonella* Typhimurium resulted in an increase in size, significant increase in the availability of nutrients, and reduced expression of certain interleukins such as IL-22, which is a crucial interleukin or mediator to maintain the gastrointestinal tract integrity and tissue generation (Bouwhuis et al. 2017). Seaweed extract, obtained from *Laminaria* sp., containing laminarin and fucoidan increased immunoglobulin G (IgG), immunoglobulin A (IgA) in sow’s colostrum and serum IgG in piglets (Leonard et al. 2012). Also, extracts from green seaweeds – *Ulva armoricana* containing sulfated polysaccharide increased IgG in sow’s blood and colostrum and the level of total IgA in milk (Bussy et al. 2019). Antioxidant enzymes (CAT and SOD) and immune-related activities (lysozyme, anti-protease, and myeloperoxidase) were higher in *Sargassum* sp. (2.5% and 5%) groups compared with control in intestinal mucus, several tissues, and serum of goat (Angulo et al. 2020). The use of seaweeds in goat diets is a good strategy to strengthen the antioxidant system and stimulate immunity, which helps to control pathogens (Angulo et al. 2020). Overall, dietary supplementation of seaweeds (*Sargassum* spp.) and their derivatives enhanced the immune responses and antioxidant capabilities (Telles et al. 2018).

Interesting research was conducted by Vizzari et al. (2021), who evaluated the effect of dietary seaweed extract (containing polysaccharides from *Laminaria digitata* and *Laminaria hyperborea*) on male-rabbit semen. It was found that after 90 days of the experiment, a decrease in aspartate aminotransferase (AST) and increase in alanine aminotransferase (ALT) and glutathione peroxidase (GPX) activities, ferric reducing ability of seminal plasma were observed with a dietary mixture of plant polyphenols and seaweed extract. Based on these results, it can be reported that natural polysaccharides extracted from brown seaweeds plus hydroxycinnamic acids, phenolic acid, flavonoids, and tannins from plant extracts mix (0.3 and 0.6%) didn’t have any adverse effect on the male rabbit reproductive parameters, but improved the animal antioxidant status, enhanced the antioxidant capability of the seminal plasma and can have a positive impact on growth, health, and development of animals.

The second most studied macroalga in animal nutrition is *Ascophyllum nodosum*. This seaweed was used as a feed additive for pigs and participated in pathogen reduction (Allen et al. 2001; Gardiner et al. 2008). Ford et al. (2020b) tested *in vitro* the antimicrobial potential of extracts obtained from *Ascophyllum nodosum* and *Fucus serratus* containing phlorotannins against foodborne pathogens such as *Escherichia coli* O157, *Salmonella agona*, and *Streptococcus suis*, which often colonize weaning piglets. These extracts were very effective, and for *A. nodosum* polyphenol extract, the minimum inhibitory concentration was between 1.56 and 0.78 mg/mL for all tested pathogens, whereas for *F. serratus* was 3.13 mg/mL. In the work of Bach et al. (2008), sun-dried *Ascophyllum nodosum* (Tasco-14^TM^) was administered at a dose of 20 g/kg diet of feedlot cattle and was efficient in the reduction in fecal shedding of the pathogen – *E. coli* O157:H7. In the *in vivo* conditions, Turner et al. (2002) examined the effect of *Ascophyllum nodosum* extract on the immune function of young pigs challenged with enteric disease caused by *Salmonella* Typhimurium. This extract had very little effect on growth performance and a positive effect on the immune function in pigs. Dietary supplementation with seaweeds (*Ascophyllum nodosum*) at 1.5% or 0.75% for 451 days improved the kidney function of minks infected with Aleutian mink disease virus (AMDV) with no impact on liver function and immunity (Farid and Smith 2020). No differences among groups were observed for antibody titer determined by the counter-immunoelectrophoresis, total serum protein, globulins, albumin, gamma-glutamyl transferase and alkaline phosphatase activities, but blood levels of creatine and urea were lower in the 1.5% *Ascophyllum nodosum* supplemented group than in the unsupplemented group (Farid and Smith 2020).

The high content of antioxidants in *A. nodosum* is responsible for the improved antioxidant status and immune functions in animals (Allen et al. 2001; Turner et al. 2002; Saker et al. 2004). In addition, *Ascophyllum nodosum* has a positive effect on the gut microbiota, translating into increased immunity (Gardiner et al. 2008; Dierick et al. 2009). In animals, modulation of gut microbiota can stimulate immunity and decrease the risk of diarrhea (Gardiner et al. 2008). According to this research, the supplement of *A. nodosum* can increase nutrient digestibility, improve nutrient availability from the gastrointestinal tract, stimulate the immune system and promote a healthy intestinal flora. When fed with a mixture of laminarin and fucoidan reduce in the growth rate appeared, but feeding purified laminarin emerged to improve health and productivity. Moreover, pigs fed with a laminarin and fucoidan enriched diet also reported increased growth productivity and good health (Gardiner et al. 2008).

When considering poultry research, the addition of *A. nodosum* extract to the broilers diet had beneficial effects on the gastrointestinal tract's integrity. It reduced the infection rates of newly born chickens colonized with *Campylobacter jejuni* in the cecum (Sweeney et al. 2016). Dietary addition of marine brown seaweeds (sodium alginate oligosaccharides) significantly reduced the mortality rate and increased body weight gain of chickens diseased with *Salmonella* Enteritidis (Yan et al. 2011). In layer diets, the use of red seaweeds (*Chondrus crispus* and *Sarcodiotheca gaudichaudii*) as prebiotics could enhance the productivity, criteria of eggs, and gut status as well as short-chain fatty acids (acetic acid, propionic acid, *n-*butyric acid, and butyric acid) in eggs (Kulshreshtha et al. 2014). In broiler chicken rations, Choi et al. (2014) found positive impacts on growth rate and immune response when used by-products of seaweed fusiforme and brown seaweed.

Coronavirus disease 2019 (COVID-19), caused by severe acute respiratory syndrome coronavirus 2 (SARS-CoV-2), is mainly a disease affecting humans, and its pandemic is ongoing. However, few reports of SARS-CoV-2 infection have been reported from animals such as dogs, cats, lions, tigers, mink (Tiwari et al. 2020; Sharun et al. 2021). Dietary seaweeds contain several nutrients and components such as angiotensin-converting enzyme 2 (ACE2) inhibitory peptides, *n*-3 fatty acids, soluble dietary fibers (fucoidan and porphyran), fucosterol, fucoxanthin, phlorotannins, and some vitamins (D_3_ and cobalamin). In addition, inhibitory action on ACE2 receptor binding of SARS-CoV-2 in the host cells and anti-inflammatory activities of seaweed extracts may also be explored in the treatment of COVID-19, which is marked by a severe hyperinflammatory response that plays a key role in the severity of infection and prognosis of the disease. Both of red algae and brown algae could be potential antiviral therapeutic agents against SARS-CoV-2 and can be used to reduce the deleterious consequences of SARS-CoV-2 infection (Pereira and Critchley 2020; Rauf et al. 2021). Dietary seaweeds and their derivatives can effectively inhibit SARS-CoV-2 entry and may provide protection against COVID-19 through different mechanisms in humans and hypothetically could render protection in animals too, for which purpose further investigation and clinical trials are required to conclude their efficacy (Li et al. 2020; Tamama 2021; Kuznetsova et al. 2021; Yim et al. 2021).

Further, several extracts of well-known invasive macroalgae, *Codium fragile*, have been reported to inhibit the inflammatory cascade in human cell lines and mice models. These studies suggest that *C. fragile* extracts interrupt the release of several proinflammatory cytokines such as TNF-α, IL-1β, and IL-6 through impairing the mechanisms of a plethora of crucial inflammatory enzymes, including COX-2, iNOS, NF-κB, and MAPK (Lee et al. 2013; Lee et al. 2017). The release of inflammatory mediators, including PGE2, NO, TNF-α, and other inflammatory mediators, such as several interleukins like 1β, and IL-6, is suppressed by seaweed-derived compounds (Hwang et al. 2015, Hwang et al. 2017; Lee et al. 2016; Sadeeshkumar et al. 2017). Additionally, fucoidan, a well-known biologically active compound from *L. japonica*, has been reported to attenuate the inflammatory response through decreased pro-inflammatory cytokines such as TNF-α, IL-6, and IL-1β. The decrease in pro-inflammatory cytokines in rat hepatic ischemia-reperfusion damage was reported to attenuate via the downregulation of several important pathways, including ERK1/2, p38, and JNK MAPK (Li and Ye 2015).

Some emerging or re-emerging viral illnesses have caused significant harm to human health in recent years, owing to the continual occurrence of these diseases. During the previous two decades, the number of antiviral agents that have been approved for clinical use has expanded from five to more than thirty medications. The polysaccharides extracted from the red alga *Gelidium robustum* (formerly *Gelidium cartilagineum*) (Rhodophyta) were found to have antiviral activity in embryonic eggs when tested against influenza B or mumps viruses, as reported by Gerber et al. (1958). In large amounts, many species of sea algae contain complex structural sulphated polysaccharides, which have been demonstrated to hinder the replication of enveloped viruses, particularly those belonging to the Nidovirales family. Other compounds, including those derived from red algae (e.g., the lectin griffithsin) and other sulphated polysaccharides derived from green algae (e.g., ulvans) and brown algae (e.g., fucoidans), have been proposed as potential antiviral therapeutic agents against SARS-CoV-2 (Pereira 2018; Barre et al. 2019; Lee 2019; Rosa et al. 2019).

### 5.3. Effect of seaweed antioxidants on animals exposed to stressful conditions

Seaweeds and their compounds are also beneficial to animals exposed to stress. Saker et al. (2004) stated that natural extracts from brown seaweeds might be used to optimize the immunocompetence of stressed animals (e.g., heat-stressed lambs) kept under suboptimal production conditions. Increased antioxidant activity in animals diminishes oxidative stress and enhances tolerance to stress (Fike et al. 2001). Antioxidants contained in the biomass of *A. nodosum* (phlorotannins, vitamins – ascorbic acid, tocopherol, and pigments – carotenoids) can scavenge peroxyl radicals and may prevent or diminish oxidative stress in weaned piglets (Michiels et al. 2012). Seaweeds’ minerals with antioxidant properties – for example, zinc and selenium also protect animals against oxidant stress (Rey-Crespo et al. 2014). Wan et al. (2017) showed that alginate oligosaccharide extracted from brown seaweeds has the potential to reverse weaning-induced oxidative stress and intestinal digestive disorders in pigs. But in case of dietary inclusion of green seaweeds (*Ulva lactuca*) to the feed of lambs reared under heat stress conditions, blood constituents and antioxidant capacity were not affected by this supplementation (Abdoun et al. 2014). Seaweed powder obtained from *Sargassum latifolium* may protect sheep from heat stress (Ellamie et al. 2020), as well as stress caused by bacterial endotoxins (Ramadan et al. 2020) and alleviate their harmful effects. Seaweed’s supplementation may improve the antioxidant defense system of animals and regulate their inflammatory and thermo-respiratory responses (Ellamie et al. 2020; Ramadan et al. 2020).

### 5.4. Effect of seaweed antioxidants on the quality of animal-derived products

Seaweeds are a rich source of important elements and compounds such as iodine, protein, and sulfated polysaccharides, which are responsible for their bioactivity in the body (Tanna and Mishra 2019; Cermeño et al. 2020; Darias-Rosales et al. 2020). Polysaccharides like laminarin and fucoidan can be used as alternatives to commercial antioxidants incorporated into animal feed (Moroney et al. 2012). An additional advantage of seaweeds, used as a source of antioxidants in animal nutrition, is the production of food enriched with these ingredients. Moroney et al. (2012) showed that there is a possibility to incorporate seaweed antioxidants and polysaccharides into animal muscle through the animal's diet. Meat and meat products enriched with antioxidants can be used to produce functional food with health-promoting properties (Holdt and Kraan 2011; Michalak et al. 2011).

Marine macroalgae can improve the lipid profile – the content of polyunsaturated fatty acids (PUFA) (e.g., González-Esquerra and Leeson 2001; Carrillo et al. 2008), the color of animal-derived products (Bonos et al. 2017), and also their multielemental composition (Michalak et al. 2011; Al-Harthi and El-Deek 2012). Rey-Crespo at al. (2014) showed that green – *Ulva rigida* and brown seaweeds – *Sargasum muticum* and *Saccorhiza polyschides* could significantly improve the mineral status of animals and the content of microelements in animal products (e.g., milk, meat). Michalak et al. (2011) found that seaweeds enriched with microelements such as Zn(II), Cu(II), Co(II), Cr(III), and Mn(II) via biosorption – increased the elements content in eggs when compared to the control group. Dierick et al. (2009) demonstrated that dried and intact *Ascophyllum nodosum* rich in iodine improved not only pig health and performance but also enriched with iodine porcine tissues (*M. psoas*, *M. longissimus dorsi*, back fat, liver, kidney, heart) and blood serum.

The supplementation of *Ascophyllum nodosum* to the diet of early lactation dairy cows significantly increased the concentration of iodine in milk (without effect on animal performance), which reached the level that may be toxic to humans, particularly children (Antaya et al. 2015). High consumption of iodine can cause damages to animal and human health. The use of analyzed seaweeds at 4 g per day did not pose a health risk (Darias-Rosales et al. 2020).

The use of seaweed feed additives can also improve the quality of animal-derived products. Braden et al. (2007) showed that supplementation of *Ascophyllum nodosum* biomass (2% Tasco) to the diet of crossbred cattle increased the amount of intramuscular fat, the marbling score, and additionally may increase meat shelf life. No effect on sensory attributes and visual color of strip-loin steaks was observed. Moroney et al. (2015) examined the effect of extract from *Laminaria digitata* containing polysaccharides (laminarin and fucoidan) on quality indices of fresh pork. This extract did not affect the plasma total antioxidant status, pH of muscle *Longissimus thoracis et lumborum*, color, microbiology, and sensory parameters, enhanced the visual sensory descriptors (pinkness, whiteness, drip, overall acceptability), and reduced saturated fatty acids and decreased lipid oxidation in fresh muscle. The content of cholesterol tended to be lower in rabbit muscle fed with the diet containing *Laminaria* spp. (0.3 and 0.6%) when compared to the control group. Also, the content of retinol and α-tocopherol was improved in both muscles (*Longissimus lumborum* and *Semimembranosus*) of rabbits receiving the algal feed additives. The sensory attributes of texture were improved in both muscles with brown seaweeds compared to control (Rossi et al. 2020). The use of 0.5, 1, and 2% of *Ascophyllum nodosum* in broiler diets did not affect the total saturated, polyunsaturated, and monounsaturated (MFA) fatty acids in the breast or the thigh muscle (Bonos et al. 2017). While the birds fed with 2% *A. nodosum* had a higher content of *n*-6 (gamma-linolenic fatty acid) in the breast muscle and a lower content of *n*-9 (eicosenoic fatty acid) when compared to the unsupplemented group (Bonos et al. 2017). The positive effect of seaweed polysaccharides on enhancing meat quality can result from the health-promoting effects of gut-associated immunity (Moroney et al. 2015).

Carrillo et al. (2012) stated that seaweeds could protect polyunsaturated fatty acids accumulated in egg yolks and can increase the storage time of these eggs. Green alga – *Enteromorpha* spp. had a protective impact on the level of docosahexaenoic acid (DHA) in eggs. In addition, the brown algae had a similar impact on the level of eicosapentaenoic acid (EPA). Furthermore, the antioxidant compounds of seaweeds like phenolic compounds, carotenoids, and vitamins (A, C, and E) play an antioxidant role in eggs enriched with *n*-3 PUFAs (Carrillo et al. 2012).

The use of seaweeds in poultry diet has a positive effect on PUFA and can improve the color of meat and yolk due to the deposition of carotenoids (Herber-McNeill and Van Elswyk 1998; Saeed et al. 2021). These pigments are important and beneficial for animals and humans due to their antioxidant activities (Al-Harthi and El-Deek 2012). Zeaxanthin, fucoxanthin, and lutein are the main carotenoids of algae that improve egg quality by increasing the yolk color (Carrillo et al. 2012). In the same context, Al-Harthi and El-Deek (2012) found that fucoxanthin improved the coloring of yolks (Al-Harthi and El-Deek 2012). On the contrary, in white leghorn laying hens, Strand et al. (1998) stated that fucoxanthin as the major carotenoid in seaweed meal was not transferred to egg yolks but gave rise to fucoxanthinol sulfate, fucoxanthinol, and paracentrone. In general, the carotenoid content in yolks increased about 12–15 times compared to the control (Strand et al. 1998). The use of *Sargassum dentifebium* (brown algae) in different sources (sun-dried, autoclaved, or boiled) at levels of 3 and 6% increased the content of lutein and zeaxanthin in egg yolks as compared to control (Al-Harthi and El-Deek 2012).

## Conclusions and future prospects

The use of natural antioxidants as feed supplements can enhance not only animal’s productivity and health but also increase their ability and resistance to many stresses like diseases, stocking density, high ambient temperatures, inadequate housing systems, and others. Seaweeds as a feed/feed additive can be used in the form of intact natural biomass or in the form of extract. The different types of seaweeds play a vital role in improving the antioxidant system in the body due to their contents of biologically active molecules like phenolic compounds, flavonoids, polysaccharides, pigments, vitamins, minerals, micro-, and macroelements. Dietary bioactive compounds in seaweeds demonstrate potential to improve animal health, productivity, and welfare. Seaweeds’ polysaccharides can improve the composition of animal’s gastro-intestinal microbiota without disrupting their performance. Also, the use of seaweeds in animal diets can boost antioxidant activity, immunity, and the gut environment. Furthermore, seaweeds have antimicrobial properties; they played an essential role in reducing the prevalence of enterohemorrhagic *E. coli* on hide swabs and in fecal samples, as well as the growth of *Salmonella* spp. It can be concluded that seaweeds with antioxidant compounds used in feed improve mainly gut health and enhance the function of the immune system. Hence, due to the available evidence of health benefits related to macroalgae and their extracts, researchers are exploring their relevant biological activities in several pathological conditions. Supplementing specific extracts of seaweeds such as laminarin in the animal feed could act as immunomodulators in many clinical conditions, including viral and bacterial infections.

Moreover, in the future, there is an urgent need to explore the biological activities of specific compounds, specifically exhibiting the antioxidant properties present in seaweeds for the enhanced production of livestock via shielding farmed animals from infections and diseases. Additionally, to acquire the efficient biological active molecules from seaweeds, there is a need to develop techniques to extract and purify biomolecules from seaweeds so that the extraction methods can be incorporated to yield macroalgae extracts with higher proportions to be utilized in several *in vitro* experiments to prevent and treat several infections in animals. However, *in vitro* screening studies and *in vivo* animal studies are needed to identify potential prebiotics from seaweeds, alongside untargeted metabolomics, to decipher microbial-derived metabolites from seaweeds. Furthermore, controlled animal intervention studies with health-related endpoints to elucidate prebiotic efficacy are required.
